# High-Power Laser Therapy for Oral Lichen Planus: A Systematic Review

**DOI:** 10.3390/jcm15031084

**Published:** 2026-01-29

**Authors:** Jakub Fiegler-Rudol, Wojciech Niemczyk, Jacek Matys, Jakub Hadzik, Dariusz Skaba, Rafał Wiench, Marzena Dominiak

**Affiliations:** 1 Department of Periodontal Diseases and Oral Mucosa Diseases, Faculty of Medical Sciences in Zabrze, Medical University of Silesia, 40-055 Katowice, Poland; dskaba@sum.edu.pl (D.S.);; 2 Department of Oral Surgery, Wroclaw Medical University, 50-367 Wroclaw, Poland; jacek.matys@umw.edu.pl (J.M.);; 3 Dental Surgery Department Faculty of Dentistry, Wroclaw Medical University, 50-367 Wroclaw, Poland

**Keywords:** oral lichen planus, CO_2_ laser, Er:YAG, laser, non-invasive treatment, chronic oral conditions

## Abstract

**Background/Objectives:** Oral lichen planus (OLP) is a chronic, autoimmune-mediated mucocutaneous disorder that significantly impacts patients’ quality of life. Conventional therapies, such as corticosteroids, are often associated with side effects, prompting the exploration of alternative treatments. High-power lasers, including CO_2_ and Er:YAG lasers, have emerged as promising options due to their precision and therapeutic potential in managing OLP. This systematic review aimed to evaluate the effectiveness of high-power lasers in reducing lesion size, pain, and recurrence rates in OLP patients. **Methods**: A comprehensive search was conducted in databases such as PubMed, Scopus, Embase and Cochrane using keywords related to laser therapy and OLP. Inclusion criteria focused on randomized controlled trials and clinical studies with clear methodologies. Data from eight studies were analyzed, covering various laser types and treatment parameters. **Results**: The findings indicate that high-power laser therapy significantly reduces lesion size, pain levels, and recurrence rates compared to conventional treatments. CO_2_ lasers demonstrated superior outcomes in lesion resolution and pain relief, while Er:YAG lasers offered precision in treating localized lesions. Most studies reported minimal side effects and faster recovery times, enhancing patient satisfaction. **Conclusions**: High-power lasers, particularly CO_2_ and Er:YAG, represent a safe and effective alternative to conventional therapies for OLP, with advantages such as reduced side effects and improved patient outcomes. Future research should focus on standardizing protocols and conducting large-scale randomized trials to validate these findings and establish lasers as a reliable treatment modality for OLP.

## 1. Introduction

### 1.1. Rationale—Oral Lichen Planus

Oral lichen planus (OLP) is a chronic immune-mediated mucocutaneous disorder that affects the oral mucosa and commonly presents as reticular white striae, erythematous areas, or painful erosive and ulcerative lesions that significantly impair quality of life [1,2,3]. Although reticular OLP may remain asymptomatic, erosive forms often produce persistent burning sensations and pain, limiting patients’ ability to eat, speak, and maintain oral function. Diagnosis is typically based on characteristic clinical features supported by histopathological findings, including basal cell degeneration, a band-like lymphocytic infiltrate, and epithelial hyperkeratosis [4,5,6]. The etiology of OLP is multifactorial, yet it is widely accepted as a T-cell-mediated autoimmune disease, in which cytotoxic lymphocytes induce apoptosis of basal keratinocytes and perpetuate chronic inflammation [5,6,7]. Psychological stress, systemic comorbidities, medications, dental materials, and infectious agents also contribute to disease onset and persistence [7,8]. Conventional treatment relies primarily on topical or systemic corticosteroids, which reduce inflammation and provide symptomatic relief but are associated with adverse effects such as mucosal atrophy, opportunistic infections, systemic absorption, and limitations in long term use [9,10]. Because many patients require prolonged or repeated therapy, and because disease recurrence is common, interest has grown in alternative modalities capable of providing effective and sustained management with fewer complications.

### 1.2. Rationale—Lasers

High-power lasers have emerged as promising therapeutic options for OLP due to their ability to precisely ablate diseased epithelial tissue, reduce local inflammatory burden, and promote re-epithelialization while minimizing damage to adjacent structures. Carbon dioxide lasers, operating at 10,600 nm, have been widely used in oral soft tissue surgery because of their ability to vaporize superficial lesions efficiently and achieve excellent hemostasis with minimal thermal diffusion [11,12,13]. Erbium-doped lasers, such as the Er:YAG system, provide similarly precise ablation due to their strong absorption in water, resulting in controlled removal of affected tissue with limited collateral effects [13,14,15,16,17,18,19]. Nd:YAG lasers, with their deeper penetration and coagulative properties, have also been investigated for symptomatic relief in erosive and mixed-type OLP lesions. Evidence from clinical trials and observational studies supports the usefulness of these systems across different clinical presentations of OLP. CO_2_ lasers have demonstrated meaningful reductions in lesion size, symptom severity, and recurrence in erosive and atrophic lesions, outperforming or equaling conventional corticosteroids in several studies. Er:YAG and Nd:YAG lasers have shown benefit in improving lesion characteristics and pain scores, either alone or in combination with pharmacologic therapies, particularly in cases where conventional therapy produced limited results. Long term observational data suggest that CO_2_ laser vaporization may reduce the persistence of erosive lesions and, in some cohorts, correspond with lower rates of malignant transformation when compared with conservative care, although these findings should be interpreted cautiously due to variable follow-up durations and study heterogeneity. Systematic reviews and scoping analyses have further highlighted the value of high-energy lasers in OLP. Additional evidence suggests that erbium-based lasers, alone or in combination with adjunctive modalities such as photodynamic therapy, can improve patient comfort and may reduce recurrence in lesions considered potentially malignant, including certain forms of OLP.

In the context of this review, the term high-energy lasers refers specifically to laser systems capable of producing ablative or coagulative effects on oral mucosal tissues. These include carbon dioxide lasers operating at 10,600 nm, which achieve superficial tissue vaporization with strong hemostasis [19,20]; erbium-based lasers such as Er:YAG (2940 nm), characterized by high absorption in water and controlled ablation with minimal thermal damage [13,14]; and neodymium-doped lasers such as Nd:YAG (1064 nm), which penetrate deeper into tissues and provide significant photothermal and coagulative effects useful in managing erosive and inflammatory OLP lesions [17,20].

### 1.3. Objectives

This systematic review aims to evaluate the effectiveness of high-power laser therapy, including CO_2_, Er:YAG, and Nd:YAG systems, in the management of oral lichen planus. The objectives are to assess reported outcomes regarding lesion size reduction, pain improvement, recurrence, and safety, while examining how different laser types perform across various clinical forms of OLP. By synthesizing available clinical evidence, this review seeks to clarify the therapeutic role of high-energy lasers and identify gaps for future well-designed randomized controlled trials.

## 2. Materials and Methods

### 2.1. Focused Question

A systematic review was conducted following the PICO framework [21], as follows: In patients with oral lichen planus (Population), does treatment with high-power lasers, including CO_2_ and Er lasers (Intervention), compared to conventional therapies such as corticosteroids or no treatment (Comparison), result in greater reduction in lesion size, pain, and recurrence rates (Outcome)?

### 2.2. Search Strategy

This review has been registered with PROSPERO under the ID CRD42024617677. This systematic review was conducted following the PRISMA 2020 guidelines, with a comprehensive electronic search performed across MEDLINE (via PubMed), Embase, Scopus, and Cochrane Database covering studies published up to 31 December 2024 [22]. The search was conducted in April 2025. The search terms, detailed in Table S1, included a wide range of keywords related to high-power lasers (e.g., CO_2_, Er:YAG, Nd:YAG lasers) and their application in treating oral lichen planus. Furthermore, the authors conducted a “snowball” search to identify additional studies by examining the bibliographies of publications selected for full-text review, and Google Scholar was employed to corroborate the veracity of the cited studies. The electronic search was limited to studies published in English to comply with the inclusion criteria. The databases were searched by three authors (W.N., R.W., J.F-R.) using identical sets of search terms. Once potential studies were identified, all authors collectively reviewed them to ensure their suitability for inclusion. To collate the data from the included studies, two authors (W.N. and R.W.) conducted a collaborative literature search to gather the necessary data.

### 2.3. Outcome Measures

The primary outcomes assessed were lesion size, clinical severity, and pain intensity. Lesion size was measured either as diameter or surface area using calibrated photographs or direct intraoral measurements. Clinical severity was evaluated through standardized scoring systems such as the Thongprasom sign score and the REU index. Pain intensity was recorded using Visual Analogue or Numeric Rating Scales at baseline and follow-up. Secondary outcomes included recurrence, healing time, inflammatory biomarkers, adverse effects, and patient satisfaction. Recurrence was defined as the reappearance of symptomatic or clinically detectable lesions during follow-up. Healing time reflected the duration required for complete epithelialization of the treated area. When measured, inflammatory cytokines were quantified using laboratory assays. Adverse effects were documented based on clinical observation or patient report. Patient satisfaction was assessed through questionnaires or verbal feedback regarding comfort and treatment response.

### 2.4. Selection of Studies

This systematic review aimed to investigate the effectiveness of high-power laser therapy, including CO_2_ and Er:YAG lasers, in treating oral lichen planus. The criteria for study inclusion and exclusion are outlined in Table 1.

### 2.5. Risk of Bias in Individual Studies

During the initial phase of study selection, each reviewer independently evaluated titles and abstracts to minimize potential biases in the assessment process. Cohen’s κ test was used to measure the degree of inter-reviewer agreement [23]. Any disagreements concerning the inclusion or exclusion of studies were resolved through discussion among the authors until a consensus was achieved.

### 2.6. Quality Assessment and Risk of Bias Across Studies

Two independent reviewers, W.N. and J.F-R., conducted quality assessments of the included studies, focusing on key methodological criteria: random allocation of participants, balanced study and control groups within a 10% margin, use of a power meter to ensure accurate laser settings, clearly defined inclusion and exclusion criteria, confirmation of diagnosis through both clinical and histopathological methods, and assurance that the source of funding did not interfere with the study results. Studies scoring 3 points or less were classified as having a high risk of bias, those scoring 4–6 points as moderate risk, and those scoring 7 or more points as low risk. Any discrepancies in scoring were resolved through discussion to reach a consensus.

### 2.7. Data Extraction

After reaching a consensus on the selection of included articles, the two reviewers (J.F-R and W.N.) extracted data on the following:

Study Details: Author, year, and study design.
Participants: Sample size, gender distribution, and age range.
Treatment Types: Interventions compared (e.g., CO_2_ laser vs. corticosteroids).
Laser Parameters: Wavelength and power output of lasers used.
Outcome Measures:
Follow-up: Duration of follow-up periods.
Adverse Effects: Notable side effects or complications observed.

## 3. Results

### 3.1. Study Selection

**Figure 1:**
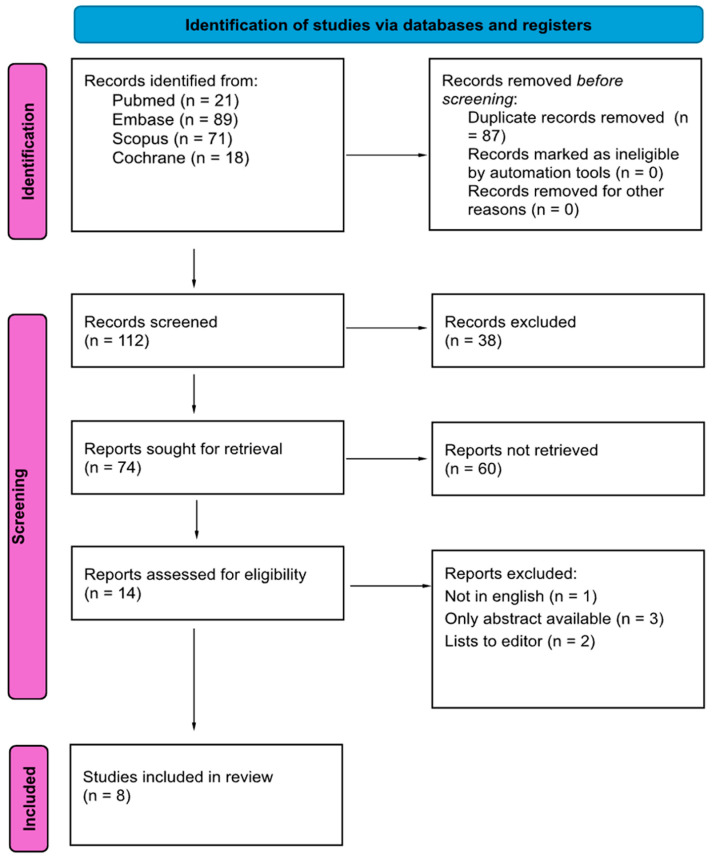
Prisma 2020 flow diagram [22].

Figure 1 provides a detailed flowchart of the research methodology, developed in alignment with PRISMA guidelines. The initial database search identified 199 articles, which were reduced to 112 after removing duplicates. Following the screening of titles and abstracts, 14 studies were deemed eligible for full-text review. Of these, 6 studies were excluded due to the following reasons: the language of the article was not English, the full text versions of the article were not available, or the article was a letter to the editor. Ultimately, the review incorporated 8 studies, all published within the past 16 years (2008–2024).

### 3.2. Risk of Bias and Quality Assessment of Evidence Results

Each study’s risk of bias was systematically assessed, with an overall rating (low, moderate, or high) assigned according to the guidelines from the Cochrane Handbook for Systematic Reviews of Interventions [24]. Among the eight included studies, none were classified as having a high risk of bias. Six studies earned eight points, while two scored seven points. Although none of the studies achieved the maximum score of nine, no exclusions were made based on quality concerns, as the omitted information was deemed non-critical for the thorough evaluation of the literature. A single point was allocated for each criterion met, whereas negative or ambiguous responses did not contribute additional points. A detailed breakdown of the criteria and the respective scores for each study is presented in Table 2.

The quality of evidence for various therapeutic interventions was evaluated using the GRADE guidelines provided by Cochrane [24], revealing significant variability (Table 3). For reduction in lesion size and pain relief, both supported by seven studies (RCTs and clinical trials), the evidence was rated as low due to moderate inconsistency and moderate imprecision [25,26,27,28,29,30,31,32]. Recurrence rates, assessed across five studies, demonstrated moderate-quality evidence, with imprecision being the primary concern [25,26,28,29,32]. Adverse effects, derived from two studies, also showed moderate-quality evidence, limited by moderate imprecision [26,32]. Patient satisfaction, informed by four studies, was rated as low quality due to moderate inconsistency and imprecision. These findings emphasize the varying levels of certainty across outcomes and highlight the need for more robust and precise research [25,28,31,32].

### 3.3. General Characteristics of the Included Studies

Out of the eight studies included, two explicitly calculated the required sample size before commencing the study, with methods such as balanced block randomization or statistical tools like G*Power (https://www.g-power.com/en/). The sample sizes varied significantly, ranging from 16 to 171 participants. The gender distribution was generally skewed towards female participants, with female-to-male ratios ranging from 3:1 to nearly equal in some studies. The mean ages of participants varied across studies, ranging from 44.8 ± 12.6 years to 62 years, with age ranges spanning from 24 to 78 years. Notably, only one study, conducted by Ibrahim et al. (2024) [26], employed a split-mouth design, while the rest followed other clinical trial methodologies. Detailed information regarding study designs, sample sizes, and demographic distributions is summarized in Table 4 and Table 5.

### 3.4. Main Study Outcomes

#### 3.4.1. Lesion Size Reduction

Across the included studies, lesion size was evaluated using linear measurements, surface area estimation, the Thongprasom sign score, or the REU index [25,26,27,28,29,30,31,32]. CO_2_ laser therapy consistently produced measurable reductions in lesion dimensions during follow-up, with several studies reporting progressive decreases in clinical severity scores after vaporization [25,26,27,28,29]. Trials comparing CO_2_ lasers with corticosteroids showed greater reductions in lesion area and REU or TSS scores in the laser group [26,29]. Nd:YAG and Er:YAG lasers also demonstrated favorable effects on lesion contraction, particularly in erosive and atrophic forms, although outcomes varied according to baseline lesion characteristics and energy parameters [27,30,31]. Long-term observational data indicated sustained lesion regression following CO_2_ laser therapy, with most treated sites maintaining clinical improvement for years [32].

#### 3.4.2. Pain Reduction

Pain was assessed using the Visual Analogue Scale or Numeric Rating Scale across all comparative and single-arm trials [25,26,27,28,29,30,31,32]. High-energy lasers produced rapid and marked improvements in pain scores, often noted within weeks of treatment [25,28]. CO_2_ laser therapy resulted in significant reductions in pain intensity and greater symptomatic relief compared with intralesional corticosteroid injections in split-mouth analyses [26]. Nd:YAG lasers produced significant decreases in pain when used alone or combined with systemic agents such as total glucosides of paeony [27,30]. Single-arm CO_2_ studies also reported consistent pain reduction over extended follow-up periods, with sustained symptom control observed up to one year or longer [28,32].

#### 3.4.3. Recurrence

Recurrence was defined as the reappearance of symptomatic or clinically detectable lesions following initial healing. Studies with follow-up intervals ranging from three months to one year showed lower recurrence rates in laser-treated groups compared with corticosteroid controls [26,29]. Split mouth comparisons demonstrated considerably longer remission periods after CO_2_ laser vaporization than with pharmacologic therapy [26]. Long term observational research, including follow-up periods extending up to eighteen years, revealed recurrence patterns similar to shorter studies, with most recurrent lesions appearing as asymptomatic or reticular forms rather than erosive lesions [32]. Er:YAG and Nd:YAG lasers likewise demonstrated reduced recurrence in multi-arm trials, though outcomes varied with lesion morphology and device parameters [31].

#### 3.4.4. Secondary Outcomes

Adverse effects were generally mild across studies and included transient postoperative discomfort and short healing times [25,26,27,28,29,30,31,32]. Epithelialization time varied with laser type, with Er:YAG treatments producing rapid re-epithelialization and Nd:YAG treatments requiring slightly longer intervals [31]. In the only study measuring inflammatory biomarkers, reductions in IL-1β, IL-6, and IFN-γ accompanied clinical improvement following high-energy laser ablation [31]. Where evaluated, patient satisfaction was high, with most patients reporting improved comfort and willingness to undergo the same treatment again [25,28,31,32].

Table 6 and Table 7 further summarize the results.

## 4. Discussion

### 4.1. Results in the Context of Other Evidence

This systematic review aimed to evaluate the effectiveness of high-power lasers, including CO_2_, Er:YAG, and Nd:YAG systems, in reducing lesion size, alleviating pain, and lowering recurrence rates in oral lichen planus. Across the eight included studies, the findings consistently indicate that high-energy laser therapy provides meaningful clinical benefits and represents an alternative or adjunct to conventional treatments, particularly for refractory or symptomatic erosive forms of OLP. The primary outcome of lesion size reduction was achieved across all laser types, with the strongest evidence supporting CO_2_ lasers. CO_2_ vaporization resulted in marked decreases in lesion dimensions and clinical severity scores, often outperforming corticosteroids in randomized comparisons [25,26,27,28,29]. Ibrahim et al. reported significantly greater improvements in REU and TSS scores with CO_2_ laser vaporization than with intralesional triamcinolone [26], while long-term observational data by Mücke et al. and Van der Hem et al. reinforced the durability of these responses [29,32]. Nd:YAG and Er:YAG lasers also contributed to lesion reduction, particularly in erosive and atrophic lesions, although effects varied depending on energy parameters and baseline presentation [27,30,31]. These results support the underlying rationale for laser ablation, as precise removal of altered epithelium diminishes the inflammatory infiltrate and promotes tissue regeneration [26,30]. Pain reduction, another key outcome, was consistently observed following high-power laser therapy. Both CO_2_ and Nd:YAG lasers rapidly decreased pain intensity as measured by VAS or NRS across all trials [25,26,27,28,29,30,31,32]. The split-mouth trial by Ibrahim et al. showed that while both CO_2_ vaporization and corticosteroids alleviated pain, CO_2_ therapy produced earlier symptomatic relief and longer-lasting comfort [26]. Nd:YAG lasers yielded comparable benefits even in the absence of adjunctive pharmacologic therapy [27], and combination protocols integrating Nd:YAG with total glucosides of paeony demonstrated enhanced analgesic response [30]. Sustained decreases in pain scores over long follow-up periods further underscore the symptomatic value of high-energy laser treatment [28,32]. Recurrence, a clinically significant concern in OLP, occurred less frequently following laser therapy than with conventional treatments. CO_2_ vaporization produced longer remission intervals and significantly reduced recurrence rates compared with corticosteroids, as shown in the split-mouth trial by Ibrahim et al. [26]. Observational data spanning up to eighteen years demonstrated that most recurrences following CO_2_ laser treatment were mild, reticular, and often asymptomatic [29,32]. Er:YAG and Nd:YAG lasers also showed promise in lowering recurrence, though findings were influenced by lesion morphology, laser mode, and patient adherence [31]. These results align with broader observations from scoping and systematic reviews suggesting that high-energy lasers can mitigate recurrence and maintain extended disease control [33,34,35,36,37,38]. Safety and tolerability outcomes were uniformly favorable. Adverse effects were mild and transient across all studies, typically limited to temporary discomfort or short healing intervals [25,26,27,28,29,30,31,32]. Healing time varied by wavelength, with Er:YAG generally enabling faster epithelialization than Nd:YAG [31]. Tarasenko et al. demonstrated reductions in inflammatory cytokines (IL-1β, IL-6, IFN-γ) following laser treatment, providing biological evidence of reduced inflammatory activity and correlating with symptomatic improvement [31]. Patient satisfaction, where assessed, was consistently high, reflecting the appeal of a minimally invasive treatment that avoids the systemic side effects associated with repeated corticosteroid use [25,28,31,32]. The discussion of laser therapy must also consider implications for malignant transformation. As OLP is classified as an oral potentially malignant disorder, long-term oncologic safety is essential [7]. Although only a minority of studies addressed this outcome directly, the available data are reassuring. Mücke et al. observed a lower frequency of squamous cell carcinoma among patients treated with CO_2_ vaporization compared with those receiving only symptomatic management, though heterogeneity in follow-up complicates definitive interpretation [29]. Similarly, Van der Hem et al. reported no malignant progression at treated sites throughout an average of eight years of observation [32]. While these findings suggest that laser therapy does not increase malignant risk and may, in some cases, reduce it, the evidence remains insufficient to draw firm conclusions. The broader literature supports the findings of this review. Prior analyses have consistently highlighted the advantages of CO_2_ and erbium-based lasers in reducing symptom burden, lowering recurrence, and improving healing characteristics in OLP [33,34,35,36,37,38,39,40,41]. These complementary findings reinforce the clinical potential of high-energy lasers and align with the results reported here. Overall, the evidence indicates that high-power laser therapy achieves the key objectives of reducing lesion size, improving pain, and decreasing recurrence in OLP while maintaining a favorable safety profile. The precision and selective action of laser devices provide therapeutic advantages consistent with contemporary principles of minimally invasive intervention. However, variability in protocols, small sample sizes, and limited long term comparative data highlight the need for more standardized and robust clinical trials to further define optimal treatment parameters and evaluate outcomes such as malignant transformation and long-term remission durability.

### 4.2. Limitations of Evidence

An important limitation of this review is the lack of homogeneity in the study designs, laser protocols, and outcome measures. Some studies, such as Agha-Hosseini et al. (2012), employed randomized designs, while others, including Van Der Hem et al. (2008), were non-randomized, increasing the risk of selection bias [25,32]. Additionally, the follow-up periods varied widely, ranging from weeks to several years, complicating comparisons of long-term efficacy. The measurement of outcomes also lacked standardization, relying primarily on subjective metrics like the VAS for pain and the Thongprasom sign score, which, while valuable, are less precise than imaging or histopathological evaluations. Few studies provided detailed descriptions of laser usage, for example, including whether it was applied in contact mode [25,27,29]. Another notable limitation is the small sample sizes across the studies [25,26,27,28,29,30,31,32]. This limitation reduces the statistical power of the findings and increases the risk of random variation influencing results. Furthermore, none of the studies included blood-based biomarkers or advanced imaging to objectively confirm lesion healing, which could have strengthened the findings. The variability in lesion types and locations, ranging from reticular to erosive forms, further complicates the generalizability of the results. Moreover, while lasers generally demonstrated fewer side effects than corticosteroids, the long-term risks, such as malignant transformation, were not adequately assessed in most studies. For instance, Mücke et al. (2015) explored the potential for squamous cell carcinoma transformation but reported inconsistent results due to the varied follow-up durations [29]. Despite these limitations, the findings suggest that high-power laser therapy offers substantial benefits over conventional treatments. Its minimally invasive nature reduced postoperative discomfort, and ability to achieve localized treatment without systemic side effects aligns with the principles of modern dentistry. The inconsistencies in study design and outcomes highlight the need for future research.

### 4.3. Limitations of Review

A notable limitation of this study is the narrative nature of synthesizing results, which the authors opted not to perform due to a lack of homogeneity among articles, due to significantly different protocols used. The variation in study groups across the reviewed research may have introduced bias, potentially impacting the objective evaluation of the effectiveness of the studies’ lasers. Given the limitations, the authors chose to provide a comprehensive summary of all available trials on the topic, allowing future researchers to identify and address any critical gaps overlooked in individual studies. However, the primary limitation of this review is the small number of randomized trials included. Conducting a systematic review and meta-analysis with a larger pool of relevant studies will be essential to strengthen future research. Interpretation of split-mouth study designs requires care, as intralesional corticosteroid administration, although primarily acting locally, may still undergo partial systemic absorption. This effect can reduce the independence of treatment outcomes between the two sides of the mouth and introduces the possibility of crossover influence. Such systemic diffusion may therefore bias comparisons between laser therapy and corticosteroid treatment in split-mouth trials, and acknowledging this limitation strengthens the interpretation of the resulting data.

### 4.4. Implications

Future studies should address these limitations by employing standardized protocols, including predefined laser parameters, consistent follow-up durations, and advanced diagnostic tools such as imaging and histopathology. Large-scale, multicenter randomized controlled trials are essential to validate the efficacy of laser therapy and ensure its reliability. Additionally, exploring the combination of laser therapy with pharmacological treatments or regenerative techniques could provide insights into optimizing patient outcomes. While the results of this review are promising, the heterogeneity of study designs, small sample sizes, and short follow-up periods prevent definitive conclusions. Nonetheless, the evidence strongly supports the potential of high-power laser therapy, particularly CO_2_ lasers, in the management of OLP. Future research should focus on addressing these gaps to fully establish lasers as a reliable and effective treatment modality for this chronic condition.

## 5. Conclusions

This systematic review evaluated the effectiveness of high-power laser therapy, including CO_2_, Er:YAG, and Nd:YAG systems, in reducing lesion size, alleviating pain, and limiting recurrence in oral lichen planus. Across the included studies, high-energy lasers consistently achieved clinically meaningful reductions in lesion severity and pain intensity, often with faster improvement and longer remission than conventional corticosteroid therapy. Recurrence rates were generally lower following CO_2_ vaporization than with pharmacologic treatment, and most relapses observed in long-term follow-up were mild and asymptomatic. Safety outcomes were favorable, with minimal adverse effects and predictable healing profiles across laser types. Although these findings support the therapeutic potential of high-power lasers for managing symptomatic or refractory OLP, the evidence base remains constrained by heterogeneity in study design, small sample sizes, and variability in laser parameters. Consequently, while the existing studies indicate that high-power lasers are a promising treatment modality aligned with contemporary minimally invasive approaches, definitive conclusions about their comparative superiority and long-term oncologic safety cannot yet be drawn. Future research should prioritize standardized treatment protocols, consistent outcome measures, and adequately powered randomized trials to clarify the optimal indications and long term benefits of laser therapy. By addressing these gaps, forthcoming studies may firmly establish high-power lasers as a reliable, evidence-based option for improving clinical outcomes in patients with oral lichen planus.

## Figures and Tables

**Table 1:** Selection criteria for papers included in the systematic review.

Inclusion Criteria	Exclusion Criteria
Randomized controlled trials	Case reports/Case series
Non-randomized controlled trials	Narrative reviews
Full text available	Systematic reviews
Human studies	Meta-analysis
English language	Non-English language publications
Patients aged ≥ 18 years	Letters to Editor
Non-smoking patients	Animal studies
Low or moderate risk of bias	Studies on smoking patients
	Conference papers

**Table 2:** Risk of Bias assessment.

Study								
Criteria	Agha-Hosseini et al. (2012) [25]	Ibrahim et al. (2024) [26]	Khater and Khattab (2020) [27]	Matsumoto et al. (2019) [28]	Mücke et al. (2015) [29]	Qi et al. (2017) [30]	Tarasenko et al. (2021) [31]	Van Der Hem et al. (2008) [32]
Random allocation	1	1	0	0	0	1	1	0
Balanced study groups (+/− 10%)	1	1	0	1	0	0	1	0
Power meter used	0	1	0	1	0	0	1	1
Inclusion/exclusion criteria clearly defined	1	1	1	1	1	1	1	1
Both clinical and histopathological diagnosis	1	1	1	1	1	1	1	1
Source of funding not interfering with the results	1	1	1	1	1	1	1	1
Total	5/6	6/6	3/6	5/6	3/6	4/6	6/6	4/6
Risk of bias	Low	Low	Moderate	Low	Moderate	Moderate	Low	Moderate

**Table 3:** GRADE Evidence quality assessment for therapeutic intervention.

Outcome	Number of Studies	Study Design	Risk of Bias	Inconsistency	Indirectness	Imprecision	Publication Bias	Overall Quality	Certainty
Reduction in lesion size [25,26,27,28,29,30,31,32]	7	RCTs, Clinical Trials	Low	Moderate (−1)	Not serious	Moderate (−1)	Not serious	Low	⊕⊕◯◯
Pain reduction [25,26,27,28,29,30,31,32]	7	RCTs, Clinical Trials	Low	Moderate (−1)	Not serious	Moderate (−1)	Not serious	Low	⊕⊕◯◯
Recurrence rates [25,26,28,29,32]	5	RCTs, Clinical Trials	Low	Not serious	Not serious	Moderate (−1)	Not serious	Moderate	⊕⊕◯
Adverse effects [26,32]	2	RCTs, Clinical Trials	Low	Not serious	Not serious	Moderate (−1)	Not serious	Moderate	⊕⊕⊕◯
Patient satisfaction [25,28,31,32]	4	RCTs, Clinical Trials	Low	Moderate (−1)	Not serious	Moderate (−1)	Not serious	Low	⊕⊕◯◯

**Table 4:** A general overview of the studies.

Author and Year	Country	Study Design	Split-Mouth
Agha-Hosseini et al. (2012) [25]	Iran	Prospective clinical trial	No
Ibrahim et al. (2024) [26]	Syria	Randomized controlled trial	Yes
Khater and Khattab (2020) [27]	Egypt	Clinical trial	No
Matsumoto et al. (2019) [28]	Japan	Single-arm intervention study	No
Mücke et al. (2015) [29]	Germany	Prospective clinical study	No
Qi et al. (2017) [30]	China	Randomized clinical trial	No
Tarasenko et al. (2021) [31]	Russia	Randomized clinical trial	No
Van Der Hem et al. (2008) [32]	Netherlands	Clinical study	No

**Table 5:** Characteristics of patients by study.

Author/Year	Sample Size Calculation	Patients	Sex	Age (Years)		
			Female	Male	Mean (±SD)	Range
Agha-Hosseini et al. (2012) [25]	Balanced block randomization, pilot study for dose determination	28	21	7	50.7	Not provided
Ibrahim et al. (2024) [26]	Calculated using G*Power, significance level 0.05, power 0.95	16	10	6	44.8 ± 12.6	32–57
Khater and Khattab (2020) [27]	Not specified	24	22	2	52 ± 14.9	24–65
Matsumoto et al. (2019) [28]	Not specified	16	14	2	62	46–75
Mücke et al. (2015) [29]	Not specified	171	87	84	52.43 ± 12.4	30–70
Qi et al. (2017) [30]	Randomized with a control group, method not specified	60	41	19	51.34 ± 10.07	41–78
Tarasenko et al. (2021) [31]	Not specified	75	59	34	Not provided	Not provided
Van Der Hem et al. (2008) [32]	Not specified	21	13	8	52.3	34–62

**Table 6:** Detailed characteristics of the studies included in this review.

Author/Year	Treatment Groups	Evaluation	Main Results	Follow-Up Period
Agha-Hosseini et al. (2012) [25]	CO_2_ laser (study group) vs. PBMT control group	Changes in lesion size, pain (VAS), and clinical response score at 2 weeks, 1, 2, and 3 months	No significant difference in baseline lesion sizes was found between the groups (PBMT: 3.2 cm; CO_2_ laser: 3.1 cm). However, lesion size reduction was significantly higher in the PBMT group at all follow-up stages (*p* < 0.05). Clinical response scores at baseline were similar in both groups, but PBMT patients showed greater improvement in clinical signs at follow-ups. In the CO_2_ group, 85% of patients showed partial to complete improvement, while 100% of PBMT patients showed such improvement. Pain reduction was observed in both groups, with significantly greater symptomatic relief in the PBMT group throughout follow-ups (*p* < 0.05).	3 months
Ibrahim et al. (2024) [26]	CO_2_ laser vaporization (study group) vs. Triamcinolone acetonide injection (control group)	REU score for lesion severity, TSS, VAS for pain, lesion area reduction at weeks 0, 4, 9	At the start, there were no significant differences between the two groups in REU, TSS, VAS scores, or lesion diameter. By the midpoint of the study, the CO_2_ group showed significant improvements in these measures (*p* < 0.001), while no significant changes were observed on the TA side. By the end of treatment, both groups demonstrated significant improvements (*p* < 0.01), though the CO_2_ group had greater improvements in REU, TSS scores, and lesion area compared to the TA group (*p* = 0.001, 0.002, and 0.048, respectively). However, there was no significant difference in VAS scores between the two groups (*p* = 0.54). During the 9-month follow-up, 75% of lesions recurred on the TA side, compared to 31.3% on the CO_2_ side, with a significant difference (*p* = 0.016). The mean time to recurrence was longer on the CO_2_ side (7.2 months) than on the TA side (3.92 months), also a significant difference (*p* = 0.034). Recurrence on the CO_2_ side was mostly reticular and asymptomatic, whereas 66.7% of recurrences on the TA side were symptomatic.	9 months
Khater and Khattab (2020) [27]	Nd:YAG laser therapy (study group) vs. no treatment (control group)	Thongprasom sign score and VAS for pain before and after treatment	Before treatment, 75% of patients reported severe oral discomfort (VAS score 3), and 25% had a VAS score of 2. After therapy, all patients experienced symptom relief, with 66% reporting mild discomfort (VAS score 1) and four patients reporting no pain (VAS score 0). The Wilcoxon matched-pairs test showed a significant reduction in pain after laser therapy (*p* = 0.0005). Initially, 59 lesions were recorded, with 45.7% showing erosive destruction (scores 4 and 5). After Nd laser treatment, 59.3% of lesions showed clinical improvement. By the end of the treatment, 33.3% of lesions had complete improvement. The Thongprasom sign scores significantly decreased after therapy (*p* < 0.0001). All patients showed some degree of improvement, with most experiencing moderate recovery, and one patient achieving complete remission with no pain and normal mucosa.	1 month
Matsumoto et al. (2019) [28]	CO_2_ laser vaporization (single-arm study)	NRS, TSS, lesion size reduction	ORL across 18 sites was analyzed. All patients initially underwent conservative treatment. 7 patients with 7 sites showed symptom improvement or declined laser treatment and were excluded from the laser treatment group. Laser irradiation was applied to nine patients at 11 sites. By day 7 post-irradiation, the NRS score had decreased at 5 of the 11 sites (45.5%) compared to pre-irradiation levels. At 1 month, 3 months, and 1 year post-irradiation, all 11 sites (100%) showed NRS score reductions. The Thongprasom sign score (TSS) decreased at 8 sites (72.7%) after 1 month, and at least 9 sites (81.8%) from 3 months to 1 year. Complete recovery (NRS and TSS scores of zero) was achieved in three sites (27.3%) after 1 year. Statistical analysis using Wilcoxon’s signed-rank test indicated that both NRS and TSS scores were significantly lower (*p* < 0.05) at 1 and 3 months (short-term), and at 6 months and 1 year (mid-long-term) compared to pre-irradiation scores. No malignant transformation was observed during the study.	1 year
Mücke et al. (2015) [29]	CO_2_ laser treatment (study group) vs. symptomatic treatment (control group)	Incidence of SCC transformation, symptomatic relief (pain management)	In the study cohort, the duration of the disease was less than 1 year in 30.9% of patients, 1–5 years in 67.3%, and more than 5 years in 1.8%. Lesions were most common in the buccal mucosa (56%), followed by the gingiva (19%), tongue (14%), and floor of the mouth (11%). Lesions were bilateral in 51% of patients. Of the 171 patients, 60.2% received symptomatic conservative treatment, while 39.8% underwent continuous defocused CO_2_ laser treatments. Following laser vaporization, 38.2% of patients experienced recurrence of erosive lesions, while 61.8% did not have further erosive lesions, although some still exhibited reticular OLP in the oral cavity. In the group receiving conservative treatment, 87.4% still had active erosive lesions, and 12.6% had no further erosive OLP but displayed reticular lesions. A total of 16 patients (9.4%) developed oral SCC, with 2 patients (2.9%) in the laser treatment group and 14 patients (13.6%) in the conservative treatment group.	2–6 years (varied by patient) 42.65 months (average)
Qi et al. (2017) [30]	Nd:YAG laser + TGP (study group) vs. TGP alone (control group)	VAS for pain, clinical sign score for lesion size	After three months of treatment, both groups showed improvements in VAS scores and physical signs. However, the observation group, which received a combination of TGP capsules and local Nd laser irradiation, had a significantly better VAS score compared to the control group. The effective rate in the treatment group was 82.1%, significantly higher than the control group (*p* < 0.05). No serious adverse reactions were observed during treatment. The study showed a significant reduction in signs and symptoms after treatment in both groups, but the observation group had better overall clinical efficacy. In contrast, the group taking only TGP capsules had an effective rate of 53.1%. Overall, the combination of TGP and Nd laser treatment was more effective than TGP alone in improving clinical outcomes.	3 months
Tarasenko et al. (2021) [31]	Er:YAG (study group 1), Nd:YAG (study group 2), Er:YAG + Nd:YAG (study group 3), scalpel surgery (control group)	IL-1β, IL-6, IFN-γ levels, pain levels, and time of epithelization	No patients were lost during the first and second follow-ups, but by the third follow-up, 1 control and 7 intervention patients were lost due to OLP exacerbation. Additionally, 10 patients were excluded for using analgesics after scalpel excision. The mean lesion size varied across groups: 2.23 cm^2^ in the Er:YAG group, 3.25 cm^2^ in the Nd:YAG group, 4.17 cm^2^ in the Er:YAG + Nd:YAG group, and 2.37 cm^2^ in the control group. Lesions were primarily located on the tongue and buccal plane (49%), followed by the palate and alveolar process (both 15%), mouth floor (11%), and lips (10%). The average ablation time was approximately 12 min for Er:YAG and 5.5 min for Nd:YAG.	2 years
Van Der Hem et al. (2008) [32]	CO_2_ laser evaporation (single-arm study) No control group	Pain relief, recurrence rate, and long-term symptom management	A total of 39 idiopathic lesions were treated with CO_2_ laser evaporation due to patient complaints. Most lesions (74%) were painful when consuming spicy foods, 7% were spontaneously painful, and 19% had pain that fluctuated over time. The clinical classification included 33% erosive/ulcerative lesions, 40% reticular, 16% plaque-like, and 11% unknown. The mean follow-up period was 8 years (range 1–18 years). After CO_2_ laser treatment, 62% of lesions showed no pain and no recurrence, while 38% had clinical recurrence. Of these, 6 lesions were painful and required retreatment with CO_2_ laser evaporation, which resolved the pain. Nine recurrent lesions were painless. All lesions healed completely within 3 weeks after treatment or retreatment. Some patients experienced new painful lesions at previously untreated locations in the mouth during follow-up. In one case, galvanic irritation was suggested as a potential etiological factor.	1–18 years (mean 8 years)

VAS (Visual Analogue Scale), PBMT (Photobiomodulation Therapy), CO_2_ laser (Carbon Dioxide Laser), Nd:YAG (Neodymium-doped Yttrium Aluminum Garnet laser), Er:YAG (Erbium-doped Yttrium Aluminum Garnet laser), REU (Reticular, Erosive, Ulcerative score), TSS (Thongprasom Sign Score), NRS (Numeric Rating Scale), SCC (Squamous Cell Carcinoma), TGP (Total Glucosides of Paeony), IL-1β (Interleukin-1 beta), IL-6 (Interleukin-6), IFN-γ (Interferon-gamma), ORL (Oral Lesions), TA (Triamcinolone Acetonide).

**Table 7:** Light sources’ physical parameters of studies that fulfilled the eligibility criteria.

Author/Year	Light Source	Operating Mode	Wavelength (nm)	Energy Density (Fluence) (J/cm^2^)	Power Output (mW)	Powermeter Used	Irradiation Time (s)	Output Spot Diameter (mm)
Agha-Hosseini et al. (2012) [25]	CO_2_ laser, Diode laser (PBMT)	Continuous (CO_2_), Pulsed (PBMT)	10,600 (CO_2_), 890, 633 (PBMT)	0.3–0.5 (PBMT)	2	Not specified	120	Not specified
Ibrahim et al. (2024) [26]	CO_2_ laser	Continuous defocused mode	10,600 nm	2.5	3	Yes	30	0.5
Khater and Khattab (2020) [27]	Nd:YAG laser	Q-switched	1064	1.2	0.5	Not specified	30	Not specified
Matsumoto et al. (2019) [28]	CO_2_ laser	Continuous-wave mode	10,600,000	8957–26,871	3	Yes	60	Not specified
Mücke et al. (2015) [29]	CO_2_ laser	Continuous defocused	10,600	Not specified	15	Not specified	Not provided	0.2
Qi et al. (2017) [30]	Nd:YAG laser	Pulsed	650	0.25	0.5	Not specified	10	2
Tarasenko et al. (2021) [31]	Er:YAG, Nd:YAG	Continuous and Pulsed	2940 (Er:YAG), 1064 (Nd:YAG)	Not specified	2	Yes	Not specified	Not specified
Van Der Hem et al. (2008) [32]	CO_2_ laser (Sharplan 791, Cavitron)	Defocused	10,600	150–200	15–20	Yes	Not provided	1

CO_2_ laser: Carbon dioxide laser. PBMT: Photobiomodulation therapy. Nd:YAG laser: Neodymium-doped Yttrium Aluminum Garnet laser. Er:YAG laser: Erbium-doped Yttrium Aluminum Garnet laser.

## Data Availability

Not applicable.

## Supplementary Materials

The following supporting information can be downloaded at: https://www.mdpi.com/article/10.3390/jcm15031084/s1, Table S1: Search Strategies and Results Across Databases for Studies on Laser Therapies and Oral Lichen Planus. File S2: PRISMA 2020 Checklist.

## References

[1] Raj G., Raj M. (2024). Oral Lichen Planus. StatPearls [Internet].

[2] Gupta S., Jawanda M.K. (2015). Oral Lichen Planus: An Update on Etiology, Pathogenesis, Clinical Presentation, Diagnosis and Management. Indian J. Dermatol..

[3] Hashemipour M.A., Sheikhhoseini S., Afshari Z., Nassab A.R.G. (2024). The relationship between clinical symptoms of oral lichen planus and quality of life related to oral health. BMC Oral Health.

[4] Nukaly H.Y., Halawani I.R., Alghamdi S.M.S., Alruwaili A.G., Binhezaim A., Algahamdi R.A.A., Alzahrani R.A.J., Alharamlah F.S.S., Aldumkh S.H.S., Alasqah H.M.A. (2024). Oral Lichen Planus: A Narrative Review Navigating Etiologies, Clinical Manifestations, Diagnostics, and Therapeutic Approaches. J. Clin. Med..

[5] Lavanya N., Jayanthi P., Rao U.K., Ranganathan K. (2011). Oral lichen planus: An update on pathogenesis and treatment. J. Oral Maxillofac. Pathol..

[6] Popa C., Sciuca A.M., Onofrei B.A., Toader S., Condurache Hritcu O.M., Boțoc Colac C., Andrese E.P., Brănișteanu D.E., Toader M.P. (2024). Integrative Approaches for the Diagnosis and Management of Erosive Oral Lichen Planus. Diagnostics.

[7] Manchanda Y., Rathi S.K., Joshi A., Das S. (2023). Oral Lichen Planus: An Updated Review of Etiopathogenesis, Clinical Presentation, and Management. Indian Dermatol. Online J..

[8] Borba Filla J., Fontanelli A.F., Brown M.A., Naval Machado M.A. (2016). Treatment of Symptomatic Oral Lichen Planus: A Literature Review. Pol. Przegląd Otorynolaryngologiczny.

[9] Lodi G., Manfredi M., Mercadante V., Murphy R., Carrozzo M. (2020). Interventions for treating oral lichen planus: Corticosteroid therapies. Cochrane Database Syst. Rev..

[10] Poetker D.M., Reh D.D. (2010). A comprehensive review of the adverse effects of systemic corticosteroids. Otolaryngol. Clin. N. Am..

[11] Keshava D. (2023). Laser Therapy in Oral Lichen Planus. Int. J. Sci. Res. Sci. Technol..

[12] Kaplan M., Vitruk P. (2015). Soft tissue 10,600 nm CO_2_ laser orthodontic procedures. Orthod. Pract. US.

[13] Riggs K., Keller M., Humphreys T.R. (2007). Ablative laser resurfacing: High-energy pulsed carbon dioxide and erbium:yttrium-aluminum-garnet. Clin. Dermatol..

[14] Momeni E., Didehdar M., Sarlak E., Safari M. (2022). In Vitro Effect of a High-Intensity Laser on Candida albicans Colony Count. J. Lasers Med. Sci..

[15] Farivar S., Malekshahabi T., Shiari R. (2014). Biological effects of low level laser therapy. J. Lasers Med. Sci..

[16] Misra N., Chittoria N., Umapathy D., Misra P. (2013). Efficacy of Diode Laser in the Management of Oral Lichen Planus. BMJ Case Rep..

[17] Elshenawy H.M., Eldin A.M., Abdelmonem M.A. (2015). Clinical Assessment of the Efficiency of Low Level Laser Therapy in the Treatment of Oral Lichen Planus. Open Access Maced. J. Med. Sci..

[18] Khalkhal E., Razzaghi M., Rostami-Nejad M., Rezaei-Tavirani M., Heidari Beigvand H., Rezaei Tavirani M. (2020). Evaluation of Laser Effects on the Human Body After Laser Therapy. J. Lasers Med. Sci..

[19] Mahdavi O., Boostani N., Jajarm H., Falaki F., Tabesh A. (2013). Use of Low-Level Laser Therapy for Oral Lichen Planus: Report of Two Cases. J. Dent..

[20] Mansouri V., Arjmand B., Rezaei Tavirani M., Razzaghi M., Rostami-Nejad M., Hamdieh M. (2020). Evaluation of Efficacy of Low-Level Laser Therapy. J. Lasers Med. Sci..

[21] Schardt C., Adams M.B., Owens T., Keitz S., Fontelo P. (2007). Utilization of the PICO Framework to Improve Searching PubMed for Clinical Questions. BMC Med. Inform. Decis. Mak..

[22] Page M.J., McKenzie J.E., Bossuyt P.M., Boutron I., Hoffmann T.C., Mulrow C.D., Shamseer L., Tetzlaff J.M., Akl E.A., Brennan S.E. (2021). The PRISMA 2020 Statement: An Updated Guideline for Reporting Systematic Reviews. BMJ.

[23] Watson P.F., Petrie A. (2010). Method Agreement Analysis: A Review of Correct Methodology. Theriogenology.

[24] Higgins J., Thomas J., Chandler J., Cumpston M., Li T., Page M., Welch V. (2023). Cochrane Handbook for Systematic Reviews of Interventions.

[25] Agha-Hosseini F., Moslemi E., Mirzaii-Dizgah I. (2012). Comparative Evaluation of Low-Level Laser and CO_2_ Laser in Treatment of Patients with Oral Lichen Planus. Int. J. Oral Maxillofac. Surg..

[26] Ibrahim R., Abdul-Hak M., Kujan O., Hamadah O. (2024). CO_2_ Laser Vaporisation in Treating Oral Lichen Planus: A Split-Mouth Randomised Clinical Trial. Oral Dis..

[27] Khater M.M., Khattab F.M. (2020). Efficacy of 1064 Q-Switched Nd:YAG Laser in the Treatment of Oral Lichen Planus. J. Dermatol. Treat..

[28] Matsumoto K., Matsuo K., Yatagai N., Enomoto Y., Shigeoka M., Hasegawa T., Suzuki H., Komori T. (2019). Clinical Evaluation of CO_2_ Laser Vaporization Therapy for Oral Lichen Planus: A Single-Arm Intervention Study. Photobiomodulation Photomed. Laser Surg..

[29] Mücke T., Gentz I., Kanatas A., Ritschl L.M., Mitchell D.A., Wolff K.D., Deppe H. (2015). Clinical Trial Analyzing the Impact of Continuous Defocused CO_2_ Laser Vaporisation on the Malignant Transformation of Erosive Oral Lichen Planus. J. Cranio-Maxillofac. Surg..

[30] Qi L., Wang X., Deng Q. (2017). The Erosive Oral Lichen Planus Treatment with Nd:YAG Laser Combined with Total Glucosides of Paeony. Glob. J. Med. Clin. Case Rep..

[31] Tarasenko S., Stepanov M., Morozova E., Unkovskiy A. (2021). High-Level Laser Therapy versus Scalpel Surgery in the Treatment of Oral Lichen Planus: A Randomized Control Trial. Clin. Oral Investig..

[32] Van Der Hem P.S., Egges M., Van Der Wal J.E., Roodenburg J.L. (2008). CO_2_ Laser Evaporation of Oral Lichen Planus. Int. J. Oral Maxillofac. Surg..

[33] Luke A.M., Mathew S., Altawash M.M., Madan B.M. (2019). Lasers: A Review with Their Applications in Oral Medicine. J. Lasers Med. Sci..

[34] Dalirsani Z., Seyyedi S.A. (2021). Treatment of Plaque-Like Oral Lichen Planus with CO_2_ Laser. Indian J. Dermatol..

[35] Habib T., Khondker L., Kabir H., Rahman M., Bhuiyan M.D.A., Nahar-E-Farzana S., Hossain M. (2024). Management of oral lichen planus by the application of carbon dioxide laser: A systematic review. J. Popul. Ther. Clin. Pharmacol..

[36] Mozafari H., Farhadzadeh K., Rezaei F. (2015). A Study of the Effects of CO_2_ Laser Therapy on Oral Lichen Planus (OLP). J. Appl. Environ. Biol. Sci..

[37] Huang Z., Wang Y., Liang Q., Zhang L., Zhang D., Chen W. (2015). The application of a carbon dioxide laser in the treatment of superficial oral mucosal lesions. J. Craniofacial Surg..

[38] Saibene A.M., Rosso C., Castellarin P., Vultaggio F., Pipolo C., Maccari A., Ferrari D., Abati S., Felisati G. (2019). Managing Benign and Malignant Oral Lesions with Carbon Dioxide Laser: Indications, Techniques, and Outcomes for Outpatient Surgery. Surg. J..

[39] Cloitre A., Rosa R.W., Arrive E., Fricain J.C. (2018). Outcome of CO_2_ laser vaporization for oral potentially malignant disorders treatment. Med. Oral Patol. Oral Y Cir. Bucal.

[40] Pakfetrat A., Falaki F., Ahrari F., Bidad S. (2014). Removal of Refractory Erosive-Atrophic Lichen Planus by the CO_2_ Laser. Oral Health Dent. Manag..

[41] Beulah J.M., Deepthi A., Gracelin, Murugan K., Deepak J.H. (2023). Efficacy of Carbon Dioxide Laser in Treating Oral Lichen Planus-A Scoping Review. J. Clin. Diagn. Res..

