# Supplementary material for: High-Power Laser Therapy for Oral Lichen Planus: A Systematic Review
Source: J Clin Med. 2026 Jan 29;15(3):1084. doi: 10.3390/jcm15031084 (PMC12898570; doi:10.3390/jcm15031084)
Supplement: Supplementary file 1 [file jcm-15-01084-s001.zip › jcm-4015322-Table S1.pdf]

Table S1. Search Strategies and Results Across Databases for Studies on Laser Therapies and Oral Lichen Planus

Source	Search term	Filters	N
Medline via PubMed	("eryag" OR "er yag" OR "erbium yag" OR "er, cr ysgg" OR "er cr ysgg" or "Lasers" or "solid-state lasers" or "solid state lasers" or "Er: yag" or "erbium yag" or "erbium yag laser" or "erbium-doped yttrium aluminium garnet laser" or "erbium lasers" or "Er,Cr: YSGG" or "erbium chromium yttrium scandium gallium laser" or "laser therapy" or "laser treatment" or "dental laser" or "diode laser" or "high level laser therapy" OR "high-level laser therapy" or "high energy laser therapy" OR "phototherapy" OR "vaporization" OR "carbon dioxide laser" OR "carbon dioxide lasers" OR "CO2 lasers" OR "CO2 laser" OR "laser surgery" OR "Nd-YAG lasers" OR "Nd YAG Lasers" OR "Erbium-Doped Yttrium Aluminum Garnet Lasers" OR Neodymium Doped Yttrium Aluminum Garnet Laser") AND ("Lichen Planus, Oral" OR "Lichen Planus, Oral/drug therapy")	Randomized controlled trials<br><br>Controlled trials	21
Scopus	TITLE-ABS-KEY (("eryag" OR "er yag" OR "erbium yag" OR "er, cr ysgg" OR "er cr ysgg" OR "Lasers" OR "solid-state lasers" OR "solid state lasers" OR "Er: yag" OR "erbium yag" OR "erbium yag laser" OR "erbium-doped yttrium aluminium garnet laser" OR "erbium lasers" OR "Er,Cr: YSGG" OR "erbium chromium yttrium scandium gallium laser" OR "laser therapy" OR "laser treatment" OR "dental laser" OR "diode laser" OR "high level laser therapy" OR "high-level laser therapy" OR "high energy laser therapy" OR "phototherapy" OR "vaporization" OR "carbon dioxide laser" OR "carbon dioxide lasers" OR "CO2 lasers" OR "CO2 laser" OR "laser surgery" OR "Nd-YAG lasers" OR "Nd YAG lasers" OR "Erbium-Doped Yttrium Aluminum Garnet Lasers" OR "Neodymium Doped Yttrium Aluminum Garnet Laser") AND ("Lichen Planus, Oral" OR "Lichen Planus, Oral/drug therapy")) AND (LIMIT-TO (DOCTYPE , "ar")) AND (LIMIT-TO (LANGUAGE , "English"))	Article<br>English language	71
Cochrane data base	("eryag" OR "er yag" OR "erbium yag" OR "er, cr ysgg" OR "er cr ysgg" OR "erbium yag laser" OR "erbium-doped yttrium aluminium garnet laser" OR "Er,Cr: YSGG" OR "erbium chromium yttrium scandium gallium laser" OR "diode laser" OR "high level laser therapy" OR "high-level laser therapy" OR "high energy laser therapy" OR "carbon dioxide laser" OR "CO2 lasers" OR "Nd-YAG lasers" OR "Nd YAG lasers" OR "Erbium-Doped Yttrium Aluminum Garnet Lasers" OR "Neodymium Doped Yttrium Aluminum Garnet Laser")<br>AND<br>("Lichen Planus, Oral" OR "Lichen Planus, Oral/drug therapy")	English language	18
Embase	('eryag' OR 'er yag' OR 'erbium yag' OR 'er, cr ysgg' OR 'er cr ysgg' OR 'Lasers' OR 'solid-state lasers' OR 'solid state lasers' OR 'Er: yag' OR 'erbium yag' OR 'erbium yag laser' OR 'erbium-doped yttrium aluminium garnet laser' OR 'erbium lasers' OR 'Er,Cr: YSGG' OR 'erbium chromium yttrium scandium gallium laser' OR 'laser therapy' OR 'laser treatment' OR 'dental laser' OR 'diode laser' OR 'low level laser therapy' OR 'low-level laser therapy' OR 'low energy laser therapy' OR 'phototherapy' OR 'photodynamic antimicrobial chemotherapy' OR 'pact') AND ('Lichen Planus, Oral' OR 'Lichen Planus, Oral/drug therapy')		89
